# Mental Patients in War Hospitals

**Published:** 1920-09-25

**Authors:** 


### THE EDITOR'S LETTER-BOX.

**Correspondence on all subjects is invited, but we cannot in any way be responsible for the opinions expressed by our correspondents, who must give their name and address as a guarantee of good faith, but not necessarily for publication. Correspondents are reminded that brevity of style and conciseness of statement greatly facilitate early insertion.**

### Mental Patients in War Hospitals.

*To the Editor of* The Hospital.

Sir,—The official report to the Home Secretary made by Sir Marriott Cooke and Dr. C. Hubert Bond, members of the Board of Control, on the organisation of the war hospitals should clear up many misconceptions. There are, of course, the recalcitrant few who will maintain their attitude of hostility towards everything that is done officially for the care and treatment of the insane, whether these be soldiers or civilians; but, realising the implacability of these critics, it is necessary largely to disregard their comments and to go steadily on with the work. Dispassionate consideration of the manner in which the arrangements for the care of the mentally disordered soldiers were carried out leads to the conclusion that a high degree of efficiency was reached with a minimum of friction between what might easily have been conflicting forces, the War Office and the Board of Control. The unremitting labours of Sir Marriott Cooke and Dr. Bond, seconded ably by the staffs of the various asylums from which the usual patients had been removed, resulted in a number of modern hospitals equipped in every way for the treatment of the military sick and wounded. In addition, a number of beds were set aside for those suffering from nervous and mental disorders. It is in connection with the mental cases that so much controversy has arisen. Categorical statements of consistent brutality and also of deprivation of privileges accorded to other patients have been freely uttered, and this by people whose word is supposed to carry some authority. Now, that there are occasional instances of rough handling of mental patients by those in charge of them no one will deny. That these offenders are dealt with drastically there is plenty of evidence in the shape of summary dismissals, prosecutions, etc., to prove. But no penal system is going to eradicate the trouble, which is, by the way, not by any means confined to institutions where the insane are housed. In order entirely to eliminate any possibility of such mischances the devoutly to be hoped for change in human character must take place which shall, for example, render organised brutality, such as war, impossible. It should be remembered that among the insane are to be found some of the most mischievous, most irritable, most brutal members of the community: persons whose lack of control has rendered them a danger to their neighbours and a pest to society. Inside an asylum they do not lose these characteristics; indeed, with their mental degradation they may tend to become more troublesome. In a moment of irritation—subsequent, for example, to receiving the contents of a utensil over his head—even an attendant of long experience may give way to anger. But even this does not suffice to exculpate him, nor is it held as sufficient excuse for infraction of the rules. Again, there is the type of patient who is reasonable enough to argue that, no matter what he may do, those in charge of him must not retaliate—"Because I am insane!" Theoretically it is easy enough to talk about turning the other cheek, but the critics would be the last to do it if they were aggravated in the manner that nurses and attendants are daily. There are also the struggles with patients which cannot be avoided. It is sheer folly and crass stupidity to talk about moral suasion in dealing, for example, with a homicidal epileptic. The thing to do is to get hold of him without delay, and that is a job the armchair critics would not be so ready for!

All this is just as applicable to the mental patients in the war hospitals—possibly more so. The patients were not all fine fellows, wounded heroes. Some of them were vicious criminals, social misfits, and a source of trouble to all with whom they came in contact. In the hospital they were always up to mean tricks, often annoying and aggravating their fellow-patients until it was difficult for the orderlies to keep the peace. It was often enough these people who posed as ill-used victims and made capital out of the credulity of those who are always ready to believe ill of anyone who has charge of the insane.

There seems to be some doubt as to the mental patients being allowed to participate in the amusements provided for the others, and this does not seem to be made clear in the report. As far as possible every effort was made not only to allow them to share these, but in many instances special entertainments were provided especially for them by the hospital authorities and also by kindly people who lived in the vicinity. There were, of course, many of the mental patients who were quite unfit to be accorded these privileges; but, as a rule, the medical staff were prepared to take reasonable risks so that the opportunities of those under their care should not be curtailed.

If it were only possible for the public to understand the difficulties which had to be faced in this matter there would be less tendency to carping criticism and a greater readiness to accord praise for the work done. The work of the war hospitals is practically a thing of the past, but the record—apart from certain blemishes inalienable from the stress and the hurry of the time—is one that may well give rise to legitimate pride in the breasts of those who were responsible for their organisation.—I am, yours faithfully,

